# Estimates of cancer incidence, mortality and survival in aboriginal people from NSW, Australia

**DOI:** 10.1186/1471-2407-12-168

**Published:** 2012-05-06

**Authors:** Stephen Morrell, Hui You, Deborah Baker

**Affiliations:** 1 Cancer Institute NSW Level 9, 8 Central Ave, Australian Technology Park, Australia; 2 School of Public Health and Community Medicine Samuels Building, University of NSW Randwick, Australia

## Abstract

**Background:**

Aboriginal status has been unreliably and incompletely recorded in health and vital registration data collections for the most populous areas of Australia, including NSW where 29% of Australian Aboriginal people reside. This paper reports an assessment of Aboriginal status recording in NSW cancer registrations and estimates incidence, mortality and survival from cancer in NSW Aboriginal people using multiple imputation of missing Aboriginal status in NSW Central Cancer Registry (CCR) records.

**Methods:**

Logistic regression modelling and multiple imputation were used to assign Aboriginal status to those records of cancer diagnosed from 1999 to 2008 with missing Aboriginality (affecting 12-18% of NSW cancers registered in this period). Estimates of incidence, mortality and survival from cancer in NSW Aboriginal people were compared with the NSW total population, as standardised incidence and mortality ratios, and with the non-Aboriginal population.

**Results:**

Following imputation, 146 (12.2%) extra cancers in Aboriginal males and 140 (12.5%) in Aboriginal females were found for 1999-2007. Mean annual cancer incidence in NSW Aboriginal people was estimated to be 660 per 100,000 and 462 per 100,000, 9% and 6% higher than all NSW males and females respectively. Mean annual cancer mortality in NSW Aboriginal people was estimated to be 373 per 100,000 in males and 240 per 100,000 in females, 68% and 73% higher than for all NSW males and females respectively. Despite similar incidence of localised cancer, mortality from localised cancer in Aboriginal people is significantly higher than in non-Aboriginal people, as is mortality from cancers with regional, distant and unknown degree of spread at diagnosis. Cancer survival in Aboriginal people is significantly lower: 51% of males and 43% of females had died of the cancer by 5 years following diagnosis, compared to 36% and 33% of non-Aboriginal males and females respectively.

**Conclusion:**

The present study is the first to produce valid and reliable estimates of cancer incidence, survival and mortality in Australian Aboriginal people from NSW. Despite somewhat higher cancer incidence in Aboriginal than in non-Aboriginal people, substantially higher mortality and lower survival in Aboriginal people is only partly explained by more advanced cancer at diagnosis.

## Background

Historically, Aboriginality has been unreliably and incompletely recorded in health and vital registration data collections for the most populous areas of Australia, including NSW, the most heavily populated state where one third of the Australian population and 29% of Australia’s Aboriginal people reside [1]. According the the 2006 Australian census, Aboriginal people numbered ≈148,000 in NSW and made up 2.2% of the state’s ≈ 6,800,000 population. A high proportion of NSW Aboriginal people live in urban areas in which over half the NSW population resides, but comprise a smaller fraction of the urban population than they do of populations in rural and remote areas.

Australian Aboriginal people suffer disproportionately higher morbidity and mortality than the non-Aboriginal population [2]. However the extent of illness and mortality in Aboriginal people, including from cancer, is known reliably only in the Northern Territory (NT), South Australia (SA) and Western Australia (WA), and often has been inferred for all Australian Aboriginal people from these sources. Cancer mortality for Aboriginal people in NSW has been estimated previously for 1994-2002 [3], and was found to be significantly higher than for NSW overall, by 71% in males and by 65% in females. Lung, colorectal and stomach cancer in both sexes, oesophageal cancer in males, and cervical, pancreatic and kidney cancer in females, were the main cancers responsible for the overall cancer mortality excess in NSW.

A 2010 Queensland study of Aboriginal cancer incidence and mortality for 1997-2006 found incidence overall to be 28% and 15% lower compared to all Queensland males and females respectively, but mortality was estimated to be 28% higher in males and 47% higher in females [4]. Mortality from cervical, uterine, lung and head and neck cancer was particularly elevated in Aboriginal females, and oesophageal, head and neck, liver and lung cancers were responsible for most of the excess in Aboriginal males.

Reliable estimates of cancer incidence in NSW Aboriginal people, however, are absent. This has been due predominantly to under-reporting of Aboriginal status in those parts of the health system where cancer is diagnosed and treated. From 1994 proportions of cancers with missing Aboriginal status decreased due to measures taken in the early 1990s nationally to remedy under-recording of Aboriginal status in death registration. In the mid-1990s NSW Health introduced measures to improve recording of Aboriginality in the NSW health system, particularly for hospital admissions. Consequently, Aboriginal status on cancer registration of living cases in particular in NSW has also improved, but until now the extent of the improvement has not been assessed, and reliable estimates of Aboriginal cancer incidence in NSW have yet to be produced.

The purpose of this paper then is to report: 

· (briefly) on an assessment of Aboriginal recording in NSW cancer registration data

· results from the imputation of Aboriginality in NSW cancer records with missing Aboriginal status

· reliable estimates of incidence, mortality and survival from cancer in NSW Aboriginal people.

## Methods

### Study design

Observational comparative cross-sectional study of the whole Aboriginal population of NSW with the non-Aboriginal population and with the NSW population overall, 1999-2007.

### Data and materials

**Figure 1:**
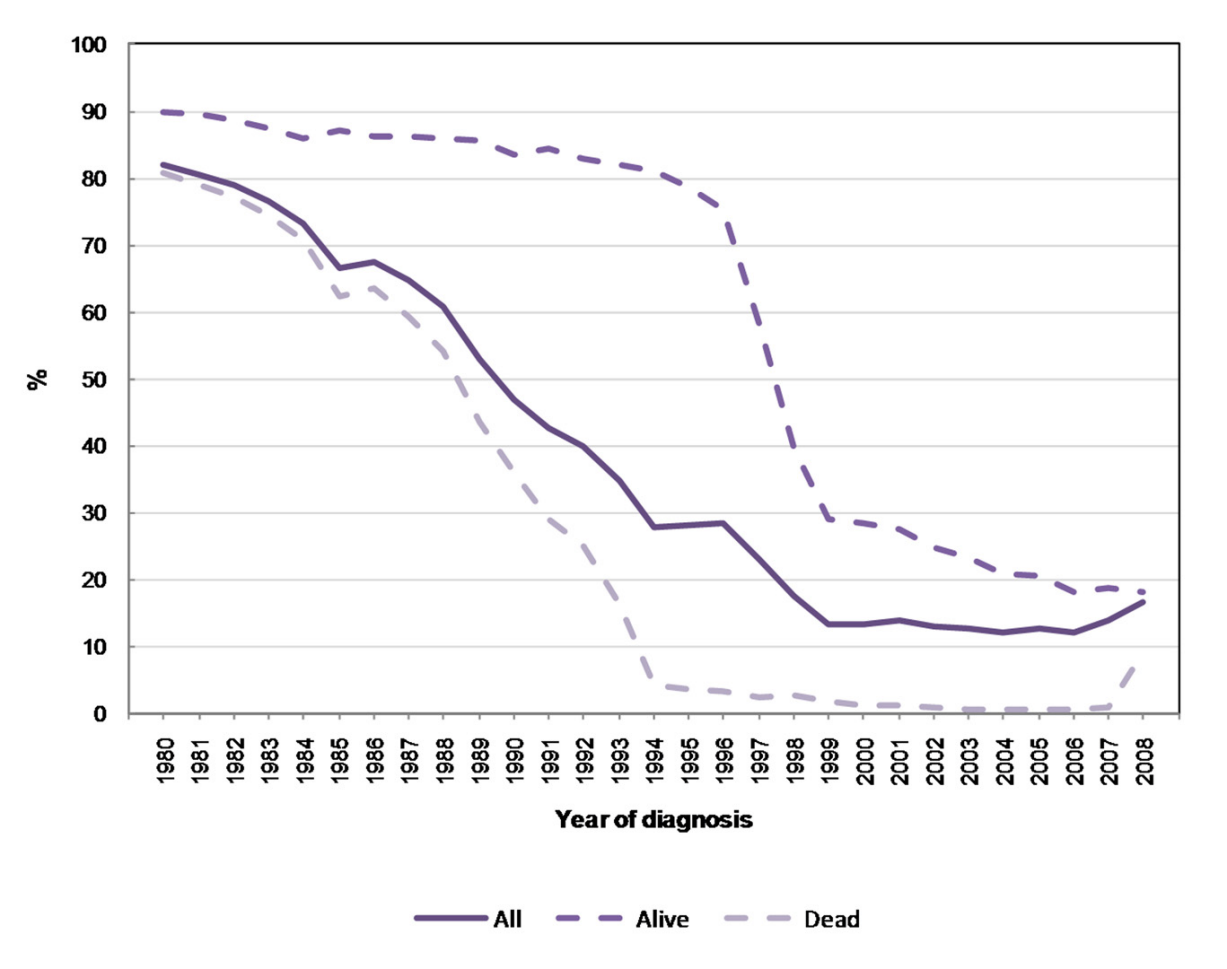
Proportion (%) of Aboriginal status missing in NSW cancer registrations by alive/dead status of cancer case.

All (de-identified) records of invasive cancers diagnosed from 1999 to 2008 held on the NSW CCR were used in this study. Cancers included in the analysis were ICD-10 codes C00-C96, D45, D46, D47.1, D47.3 and non-melanoma skin cancer (C44) was excluded. In order to estimate incidence and minimise recording bias of Aboriginal status in NSW cancer data, logistic regression modelling of cancer records with non-missing Aboriginal status was used to inform a multiple imputation (MI) approach to assigning Aboriginal status to those records where Aboriginality is missing. Cancers diagnosed from 1999 onwards were chosen for imputation and analysis since the proportions of records with missing Aboriginal status were lowest from then (Figure 1). Cancer mortality data as recorded by the CCR from 1994 onwards are considered sufficiently reliable to use, but for this paper we have estimated Aboriginal cancer mortality for 1999-2007, to inform the survival analysis of cancers diagnosed over 1999-2007.

### Analysis

Essentially, MI is a means for representing uncertainty in missing data, by producing a distribution of plausible values for a missing variable in a record, given the values of that record’s non-missing covariates. The contribution of the non-missing covariates to the distribution of missing values is based on modelling the non-missing co-variates in complete cases to predict the likely value of the variable in records where it is missing.

The key assumption of most methods for handling missing data is that of Missing Completely at Random (MCAR), which essentially means that the observations that are missing are a simple random sample of all observations. This is clearly not the case for Aboriginal status.

However, MI requires only the more relaxed assumption of Missing at Random (MAR), which means that the missing data is a random sample of all the data after adjusting for all other observed (non-missing) variables [5]. In other words, systematic differences between missing cases and the complete cases are allowed so long as all (or most) of the differences can be explained or modelled by the other observed variables. If the proportion of missingness in the records is low then multiple imputation can be used [6].

Once a model predicting values for a missing variable is constructed, Markov chain Monte Carlo (MCMC) simulation is used to create a small number of independent draws (typically 5-10) of records with a range of imputed values from the predictive distribution. Each draw represents a random sample of the missing values and is then used for multiple-imputation inference such that a number of data sets (equal to the number of draws) of ‘complete’ cases are analysed using standard statistical analyses. The results from the ‘complete’ data sets are then combined to produce inferences that account for multiple records of the same individual from the draws. The overall estimate is the average of the individual estimates, and the overall standard errors include both between and within imputation standard errors. Standard errors for the missing variable reflect the uncertainty due to the missing values, leading to valid statistical inferences.

MI is particularly suitable for estimating Aboriginal cancer rates, as there are numerous covariates available in the NSW CCR (such as area of residence, sex, age, country of birth and clinical variables) which make it quite likely that the MAR assumption would be met. For cancer mortality, missing Aboriginal status has been very low on the NSW CCR since 1999, ranging between 0.2% and 2.8% over 1999-2007 (cf. Figure 1). Accordingly, multiple imputation was applied to cancer incidence data only.

The statistical modelling approach used to develop the imputation model for the MI procedure was logistic regression, since the missing variable is a binary quantity. No covariates other than Aboriginality had missing values, which simplified the estimation procedure considerably. Covariates included in the model were 5-year age group, sex, country of birth, cancer degree of spread at diagnosis, clinical grouping, one-year survival, Area Health Service of residence at diagnosis, and percentage of the local government area population identifying as Aboriginal in the most recent population census. Area Health Service was included to capture geographical remoteness and differences in health resources and access to health services. The logistic regression model was fitted to all cancer data with non-missing Aboriginal status for 1999-2008, treating Aboriginal status as a binary outcome (1 = Aboriginal or Torres Strait Islander, 0 = otherwise).

For the logistic regression modelling and imputation, we used Proc Logistic, Proc MI and Proc MIAnalyze in SAS/STAT^®^ software, version 9.2. Directly age-standardised cancer incidence and mortality rates were calculated using the 2001 Australian census population as the standard. Indirectly age-standardised incidence and mortality ratios were calculated using cancer incidence and mortality expected in the whole NSW male or female population, as appropriate, for 1999-2007. Cumulated risks of cancer incidence and mortality to age 75 years were calculated according to the method of Day [7]. Cox proportional hazards regression modelling was used to produce cancer-specific hazard ratios by Indigenous status by sex, adjusted for age and year of diagnosis, and cancer degree of spread at diagnosis.

A sensitivity analysis was conducted in which 0-5% random samples of all cancers with missing Aboriginal status were assigned as Aboriginal and overall Indigenous cancer incidence estimated with these added to cancers recorded as Indigenous.

The Cancer Institute NSW is the organisation with delegated authorisation to manage the NSW Central Cancer Registry on behalf of the NSW Ministry of Health. The management of this registry includes assessment, review, maintenance and enhancement of the quality of all data held in the registry. The work for this project was undertaken within this ambit. In addition, no identified individual case information was utilised. Therefore formal ethical approval was not required to undertake this work.

## Results

### Aboriginal status missing

**Figure 2:**
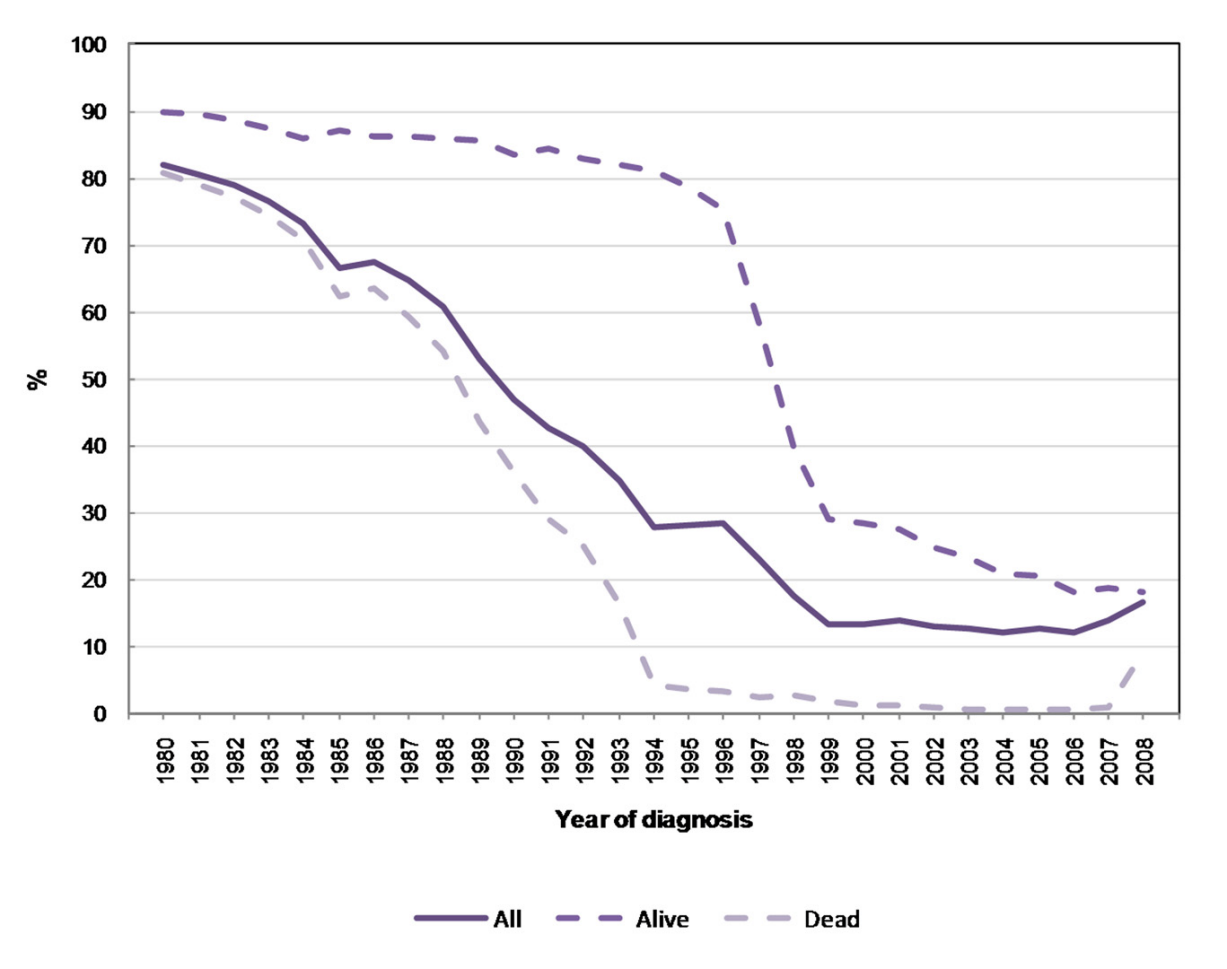
Inpatient admission episodes and proportion (%) of Aboriginal status missing in alive cancer cases, 1999-2007.

As Figure 1 indicates, Aboriginal status on cancer registration in NSW has improved considerably since the mid-1990s: for cancer deaths, missing Aboriginal status affects 1-2% of cases diagnosed since 1999 (the rise for 2008 is artefactual due to CCR death information yet to be fully updated from the Australian Bureau of Statistics). For still-living cancer cases, the proportion with unknown Aboriginality status declined sharply from 75% in 1996 to 30% in 1999 and steadily thereafter to under 20% by 2008. For all registered cancers, the proportion with missing Aboriginal status varied between 12% and 14% over 1998-2007. While there is still a substantial proportion of missing Aboriginal status in live cancers this is not so great as to preclude multiple imputation. From the perspective of care episodes, proportions of missing Aboriginal status in alive cancer cases are generally around 90% if no inpatient hospital admission occurs, but declines to 10-20% by the first hospital admission for the cancer, then to negligible levels by the fourth admission (Figure 2).

### Model used in multiple imputation

From the logistic regression model applied to cancer records with known Aboriginal status, most of the odds ratio estimates were statistically significant (Table 1). All age groups of cancer diagnosis younger than 75+ years were positively and significantly associated with Aboriginality, as were distant and regional degree of cancer spread, death within 1 year of diagnosis, percentage of Aboriginal population in the local government area of residence (at diagnosis), and non-metropolitan NSW Area Health Services with relatively high Aboriginal population compositions. Significant negative predictors included country of birth outside Australia, and most cancer clinical groupings (using respiratory cancer as the referent). The concordance from the logistic regression model was 74.4% and the percentage discordant was 20.0%, leaving 5.6% tied. The resulting area under the ROC curve of 0.772 suggests that the model correctly predicts Aboriginal status in most cases.

**Table 1:** Predictors^1^ of Aboriginal status in NSW CCR registered cancers 1999-2007, from logistic regression model

Predictor	Predictor category	OR (95% CI)^2^
Age group (yr)	0 - 4	3.73 (2.48 - 5.61)
	5 - 9	4.43 (2.58 - 7.58)
	10 - 14	2.13 (1.07 - 4.24)
	15 - 19	3.45 (2.13 - 5.60)
	20 - 24	3.54 (2.34 - 5.36)
	25 - 29	3.35 (2.35 - 4.77)
	30 - 34	2.85 (2.08 - 3.90)
	35 - 39	4.05 (3.23 - 5.07)
	40 - 44	3.88 (3.20 - 4.69))
	45 - 49	3.30 (2.78 - 3.92)
	50 - 54	3.16 (2.71 - 3.69)
	55 - 59	2.55 (2.20 - 2.96)
	60 - 64	2.46 (2.14 - 2.84)
	65 - 69	1.88 (1.63 - 2.18)
	70 - 74	1.59 (1.38 - 1.85)
	75 +	1.00 (referent)
Sex	Male	0.93 (0.85-1.02)
	Female	1.00 (referent)
Country of Birth	Not Australia	0.22 (0.19-0.25)
	Unknown	0.90 (0.66-1.22)
	Australia	1.00 (referent)
Degree of Spread	Regional	1.20 (1.07-1.33)
	Distant	1.27 (1.12-1.43)
	Unknown	1.05 (0.94-1.18)
	Localised	1.00 (referent)
Clinical Grouping	Skin	0.24 (0.19-0.31)
	Head and Neck	1.11 (0.91-1.35)
	Upper Gastrointestinal	0.87 (0.74-1.01)
	Colorectal	0.54 (0.46-0.62)
	Bone and other connective tissue	0.53 (0.34-0.82)
	Breast	0.58 (0.50-0.69)
	Gynaecological	0.90 (0.74-1.09)
	Urogenital	0.56 (0.48-0.64)
	Eye	0.46 (0.21-1.00)
	Neurological	0.57 (0.42-0.76)
	Thyroid and other endocrine	0.44 (0.32-0.62)
	Lymphohaematopoeitic	0.65 (0.54-0.77)
	Ill-defined and unknown primary sites	0.64 (0.52-0.79)
	Respiratory	1.00 (referent)
One-year survival	Death within 1 year from Diagnosis	1.36 (1.25-1.49)
	Survival to 1 year from Diagnosis	1.00 (referent)
Area Health Service	Sydney South West AHS	0.74 (0.63-0.87)
	Sydney West AHS	0.86 (0.73-1.00)
	Northern Sydney and Central Coast AHS	0.59 (0.50-0.70)
	Hunter and New England AHS	1.29 (1.13-1.47)
	North Coast AHS	1.28 (1.11-1.49)
	Greater Southern AHS	1.21 (1.03-1.41)
	Greater Western AHS	1.83 (1.56-2.13)
	South Eastern Sydney and Illawarra AHS	1.00 (referent)
Percentage Aboriginal in		
Local Government Area^3^		1.13 (1.12-1.15)

^1^Co-variates simultaneously adjusted for.

^2^Odds ratio and 95% confidence interval.

^3^Of residence at diagnosis.

### Cancer Incidence

After imputation, 146 additional cancers diagnosed in 1999-2007 were assigned as Aboriginal in males (12.2% more than recorded), and 140 cases to females (12.5% more than recorded). Mean annual Aboriginal cancer incidence was estimated to be 660 per 100,000 in males and 462 per 100,000 in females, equivalent to 9% and 6% higher than all NSW males and females respectively, as measured by standardised incidence ratios (Tables 2 and 3). Head and neck (SIR = 2.05, males and 2.01, females), oesophagus (1.85 and 2.16), liver (1.67 and 2.21) and lung (1.93 and 2.43) were significantly higher in both sexes. Males also had significantly higher incidence rates of cancers of unknown primary origin (SIR = 1.63) and stomach (1.85). Females had a significantly higher incidence of cervical cancer (SIR = 2.43). Male and female Aboriginals had significantly lower incidence of melanoma (SIR = 0.44, males and 0.47 females). Incidence of prostate cancer (SIR = 0.82) was also significantly lower than for all NSW males, and thyroid cancer incidence was significantly lower in Aboriginal females (SIR = 0.54). Prostate cancer is the most commonly diagnosed cancer in Aboriginal males (145 per 100,000), followed by lung cancer (111 per 100,00) and large bowel (75 per 100,000). Breast cancer is the most common cancer diagnosed in Aboriginal females (116 per 100,000), followed by lung (69 per 100,000), and cancer of the large bowel (56 per 100,000). The cumulative risk of cancer over ages 0 to 75 years in Aboriginal males was 1 in 2.6, compared to 1 in 2.8 for non-Aboriginal males; in Aboriginal females the cumulative risk was 1 in 3.5, compared to 1 in 3.8 for non-Aboriginal females.

**Table 2:** Estimates of cancer incidence in Aboriginal males, NSW, 1999-2007, and risk comparison with non-Aboriginal males

Cancer site	Registered cases	Missing (%)	Imputed cases	Imputed incidence^3^ per 100,000 (95% CI)	SIR^1^(95% CI)	Risk^2^ to age 75, 1 in:	
						Aboriginal	Non-Aboriginal
Head and neck	96	6.4	103	37.9 (28.5-47.2)	2.05 (1.64-2.46)	34	59
Oesophagus	27	2.2	28	13.6 (7.5-19.7)	1.85 (1.15-2.54)	86	174
Stomach	46	3.1	47	25.5 (16.7-34.3)	1.85 (1.32-2.38)	54	107
Large bowel	147	6.4	158	75.4 (60.6-90.3)	1.06 (0.88-1.23)	17	17
Liver	29	2.1	30	11.7 (6.9-16.6)	1.67 (1.06-2.27)	81	172
Pancreas	32	1.1	32	17.7 (10.2-25.1)	1.53 (1.00-2.06)	87	124
Lung	200	2.1	206	111.0 (92.6-129.4)	1.93 (1.66-2.19)	12	23
Melanoma of skin	37	41.2	67	33.6 (21.3-45.9)	0.44 (0.29-0.58)	48	23
Mesothelioma	11	1.7	11	7.4 (2.4-12.5)	1.32 (0.54-2.10)	211	276
Prostate	183	20.0	239	144.5 (120.0-169.0)	0.82 (0.71-0.94)	10	8
Kidney	50	9.1	53	24.1 (15.9-32.4)	1.31 (0.95-1.66)	60	73
Bladder	30	5.3	31	23.3 (13.7-33.0)	1.07 (0.69-1.45)	105	87
Brain	20	2.8	21	5.5 (2.2-8.8)	0.74 (0.42-1.07)	262	150
Non-Hodgkin’s lymphoma	47	10.2	53	17.1 (11.4-22.7)	0.95 (0.68-1.22)	62	60
Multiple myeloma	17	6.3	18	9.1 (3.9-14.2)	1.24 (0.65-1.83)	130	180
All leukaemias	51	8.6	56	23.8 (15.0-32.5)	1.06 (0.77-1.35)	70	83
Unknown primary	59	4.7	61	30.1 (20.7-39.5)	1.63 (1.21-2.05)	47	77
Myelodysplasia	27	6.1	29	18.1 (9.7-26.4)	1.50 (0.94-2.07)	118	157
All cancers	1,201	13.6	1,347	660.2 (613.7-706.6)	1.09 (1.02-1.15)	2.6	2.8

^1^Standardised incidence ratio in relation to all NSW males.

^2^Cumulative risk.

^3^Directly age-standardised to 2001 Australian census population.

**Table 3:** Estimates of cancer incidence in Aboriginal females, NSW, 1999-2007, and risk comparison with non-Aboriginal females

Cancer site	Registered cases	Missing (%)	Imputed cases	Imputedincidence^3^ per 100,000 (95% CI)	SIR^1^(95% CI)	Risk^2^ to age 75, 1 in:	
						Aboriginal	Non-Aboriginal
Head and neck	36	8.0	39	13.1 (8.5-17.6)	2.01 (1.37-2.65)	81	193
Oesophagus	14	1.6	14	5.8 (2.4-9.1)	2.16 (1.03-3.29)	207	521
Stomach	18	3.6	19	8.2 (4.0-12.5)	1.35 (0.73-1.98)	173	253
Large bowel	110	7.7	120	56.0 (44.4-67.6)	0.95 (0.77-1.13)	29	26
Liver	15	1.7	15	6.4 (2.8-10.1)	2.21 (1.09-3.33)	213	533
Pancreas	28	1.5	29	14.4 (8.5-20.2)	1.49 (0.94-2.04)	119	166
Lung	162	3.1	167	68.7 (57.0-80.3)	2.43 (2.06-2.81)	19	44
Melanoma of skin	30	46.7	62	20.2 (12.9-27.4)	0.47 (0.31-0.62)	74	34
Breast	304	12.2	346	115.7 (101.2-130.3)	0.94 (0.84-1.05)	12	11
Cervix	65	12.4	71	19.0 (13.7-24.4)	2.43 (1.83-3.03)	69	186
Uterus	43	8.7	50	21.0 (14.2-27.8))	1.16 (0.82-1.51)	65	77
Ovary	36	4.5	38	12.0 (7.5-16.4)	1.11 (0.74-1.47)	109	112
Kidney	31	10.0	34	12.9 (7.8-17.9)	1.33 (0.87-1.80)	105	140
Brain	22	2.5	23	6.8 (3.4-10.2)	1.08 (0.63-1.53)	174	235
Thyroid	24	12.2	28	6.5 (3.7-9.3)	0.54 (0.33-0.75)	173	107
Non-Hodgkin’s lymphoma	26	11.4	33	11.2 (6.4-16.1)	0.76 (0.48-1.04)	96	87
All leukaemias	30	8.3	33	10.3 (5.5-15.0)	0.89 (0.58-1.20)	203	142
Unknown primary	44	4.1	47	18.1 (12.1-24.2)	1.29 (0.90-1.68)	73	101
Myelodysplasia	14	8.2	16	6.9 (2.9-11.0)	1.00 (0.47-1.53)	272	249
All cancers	1,117	12.1	1,257	462.2 (431.9-492.6)	1.06 (1.00-1.13)	3.5	3.8

^1^Standardised incidence ratio in relation to all NSW females.

^2^Cumulative risk.

^3^Directly age-standardised to 2001 Australian census population.

**Figure 3:**
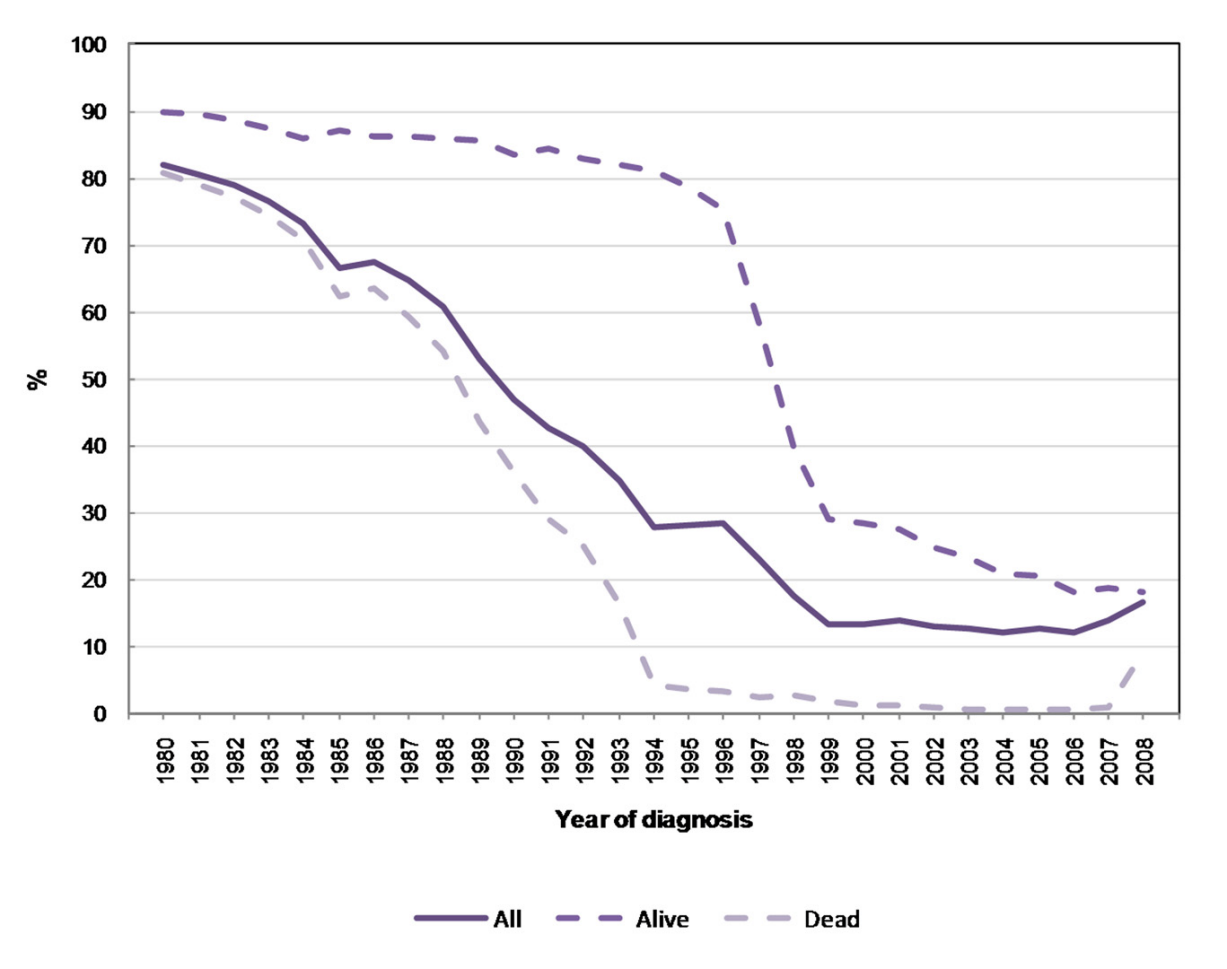
Age-specific cancer incidence and mortality, per 100,000 Aboriginal and non-Aboriginal males and females, NSW, 1999-2007.

The age distribution of cancer incidence in Aboriginal people shows most of the excess over non-Aboriginal people to be occurring in the oldest age groups, manifesting by age 70+ years (Figure 3). By age 75+ years cancer incidence in Aboriginal people is approximately 30% and 40% higher in males and females respectively.

### Cancer Mortality

Mean annual cancer mortality in NSW Aboriginal people is estimated to be 373 per 100,000 in males (from 657 deaths over 1999-2007), and 240 per 100,000 in females (from 572 deaths; Tables 4 and 5). Cancer mortality in Aboriginal people was 68% and 73% higher than in all NSW males and females respectively, as measured by standardised mortality ratios. The higher excess mortality over incidence indicates considerably lower cancer survival. Outcomes for Aboriginal males with prostate cancer are particularly poor: despite 18% lower incidence of prostate cancer than for NSW overall, Aboriginal males have 86% higher prostate cancer mortality. Mortality from cervical cancer, one of the few easily preventable, was 374% higher in Aboriginal than all NSW women, considerably surpassing the 143% excess incidence of cervical cancer (Table 5). Aboriginal mortality is substantially higher than NSW from several other key cancers including lung (SMR = 1.99, males and 2.73, females), female breast (SMR = 1.54). Even for melanoma, with standardised incidence ratios of 0.44 for males and 0.47 for females, the standardised mortality ratios were not significantly different from 1 (0.67 in males, 1.00 in females).

Lung cancer is the leading cause of cancer death in Aboriginal males (91 per 100,000), followed by prostate cancer (57 per 100,000) and large bowel (39 per 100,000) (Table 4). Lung cancer is also the most common cancer cause of death in Aboriginal females (58 per 100,000), followed by breast (36 per 100,000) and large bowel (24 per 100,000) (Table 5). The cumulative risk of cancer mortality over ages 0 to 75 years in Aboriginal males was 1 in 4.7, compared to 1 in 7.5 for non-Aboriginal males; in Aboriginal females the cumulative risk of cancer mortality was 1 in 6.5, compared to 1 in 11.1 for non-Aboriginal females.

As for incidence, the age distribution of cancer mortality in Aboriginal people shows most of the excess over non-Aboriginal people occurring in the oldest age groups, except the excess mortality manifests earlier at 60+ years (Figure 3). By age 75+ years cancer mortality in Aboriginal people is approximately 80% higher in males and 75% higher in females.

**Table 4:** Cancer mortality in Aboriginal males, NSW, 1999-2007, and risk comparison with non-Aboriginal males

		Mortality rate^1^ per 100,000 (95% CI)		Risk^3^ to age 75, 1 in:	
Cancer site	Deaths		SMR^2^ (95% CI)	Aboriginal	Non-Aboriginal
Head and neck	54	20.6 (14.5-28.0)	3.18 (2.39-4.15)	56	155
Oesophagus	25	12.9 (7.6-20.1)	2.31 (1.50-3.42)	86	241
Stomach	34	21.2 (13.5-31.1)	2.09 (1.45-2.92)	71	165
Large bowel	68	38.6 (28.0-51.2)	1.40 (1.08-1.77)	45	52
Liver	20	7.8 (4.2-12.8)	1.74 (1.06-2.68)	135	250
Pancreas	30	19.0 (11.6-28.7)	1.70 (1.15-2.42)	94	147
Lung	164	91.1 (75.3-108.8)	1.99 (1.69-2.31)	14	29
Melanoma of skin	12	8.1 (3.5-15.0)	0.67 (0.35-1.17)	174	173
Prostate	65	57.1 (42.6-74.5)	1.86 (1.43-2.37)	51	86
Kidney	19	10.5 (5.4-17.8)	1.70 (1.02-2.66)	159	254
Bladder	10	7.3 (2.8-14.6)	1.12 (0.54-2.06)	366	316
Brain	14	4.4 (1.7-8.4)	0.76 (0.42-1.28)	346	191
Non-Hodgkin’s lymphoma	18	7.9 (4.0-13.6)	1.09 (0.64-1.72)	169	176
All leukaemias	27	16.0 (9.3-25.1)	1.44 (0.95-2.10)	115	185
Unknown primary	48	25.1 (17.1-35.0)	1.86 (1.37-2.46)	57	106
All cancers	657	372.9 (338.9-409.0)	1.68 (1.55-1.81)	4.7	7.5

^1^Directly age-standardised to 2001 Australian census population.

^2^Standardised mortality ratio in relation to all NSW males.

^3^Cumulative risk.

**Table 5:** Cancer mortality in Aboriginal females, NSW, 1999-2007, and risk comparison with non-Aboriginal females

		Mortality rate^1^ per 100,000 (95% CI)		Risk^3^ to age 75, 1 in:	
Cancer site	Deaths		SMR^2^ (95% CI)	Aboriginal	Non-Aboriginal
Head and neck	18	6.8 (3.8-11.1)	3.21 (1.90-5.07)	135	608
Oesophagus	11	4.5 (2.0-8.5)	2.40 (1.20-4.29)	316	787
Stomach	17	7.9 (4.2-13.2)	1.98 (1.15-3.16)	235	433
Large bowel	49	23.6 (16.7-32.2)	1.24 (0.92-1.64)	81	87
Liver	10	4.6 (2.0-8.9)	2.19 (1.05-4.03)	260	768
Pancreas	29	15.0 (9.6-22.2)	1.82 (1.22-2.61)	118	201
Lung	134	57.8 (47.4-69.6)	2.73 (2.29-3.23)	25	62
Melanoma of skin	9	4.1 (1.6-8.2)	1.00 (0.46-1.89)	325	442
Breast	99	35.6 (28.0-44.4)	1.54 (1.25-1.88)	37	59
Cervix	31	10.9 (6.8-16.2)	4.74 (3.22-6.73)	123	663
Uterus	13	6.4 (3.1-11.5)	2.05 (1.09-3.51)	259	490
Ovary	22	8.3 (4.8-13.1)	1.35 (0.84-2.04)	157	203
Kidney	16	6.9 (3.6-11.7)	2.36 (1.35-3.82)	188	469
Brain	15	5.5 (2.7-9.7)	1.20 (0.67-1.98)	208	315
Non-Hodgkin’s lymphoma	13	5.3 (2.5-9.6)	1.00 (0.53-1.72)	213	277
All leukaemias	15	5.4 (2.5-9.6)	1.17 (0.66-1.94)	389	345
Unknown primary	35	14.4 (9.4-20.8)	1.35 (0.94-1.88)	103	138
All cancers	572	240.3 (218.5-263.5)	1.73 (1.59-1.88)	6.5	11.1

^1^Directly age-standardised to 2001 Australian census population.

^2^Standardised mortality ratio in relation to all NSW females.

^3^Cumulative risk.

### Cancer degree of spread

**Figure 4:**
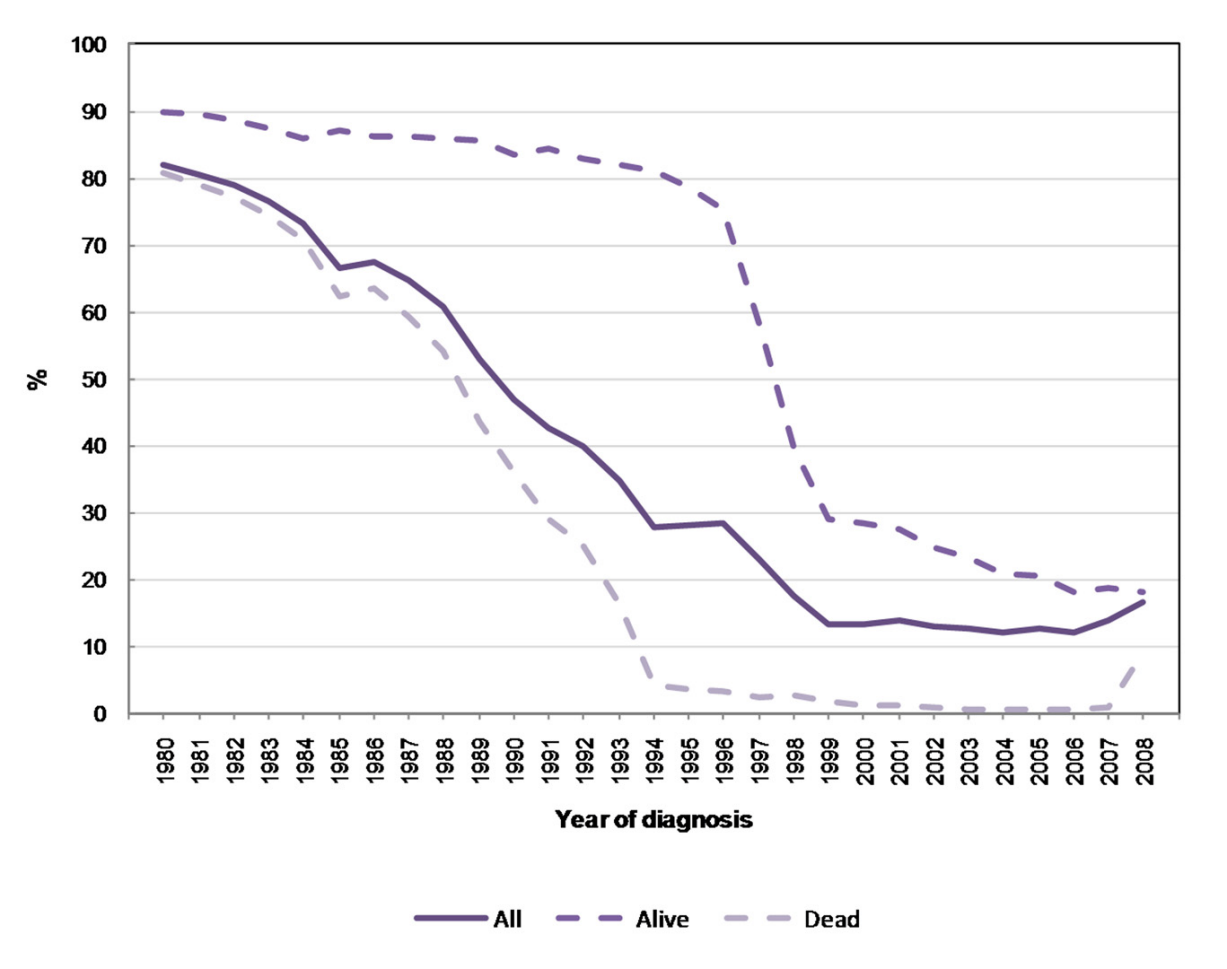
Age-standardised cancer incidence and mortality by degree of spread at diagnosis, Aboriginals and non-Aboriginals, males and females, 1999-2007.

Differences in cancer stage at diagnosis are evident but indicate only some of the sources of differences between Aboriginal and non-Aboriginal cancer mortality. Incidence of cancer with regional or distant degree of spread is significantly higher in Aboriginal males and females than non-Aboriginal counterparts, and mortality correspondingly is significantly higher (Figure 4). However, while incidence by localised cancer in Aboriginal people is somewhat lower (non-significantly), and is non-significantly higher for cancer of unknown degree of spread, Aboriginal mortality from localised and unknown degree-of-spread cancers is significantly higher. This can be seen more clearly by examining mortality to incidence (M:I) ratios. In Aboriginal males the M:I ratio for localised cancer is 0.32 compared to 0.21 in non-Aboriginal males; in Aboriginal females the M:I ratio is 0.23, compared to 0.12 in non-Aboriginal females. For regional degree-of-spread cancers, these ratios are 0.32 versus 0.17 in males, and 0.25 versus 0.14 in females; for metastatic cancer the M:I ratios are 0.49 versus 0.28 in males, and 0.32 versus 0.19 in females; and for cancer of unknown origin the M:I ratios are 0.52 versus 0.32 in males and 0.25 versus 0.16. Thus not all the excess mortality from cancer in Aboriginal people is explained by higher cancer stage at diagnosis. Mortality outcomes for Aboriginal people diagnosed with cancer of the same degree of spread are significantly and substantially worse than in non-Aboriginal people.

### Cancer-specific Survival

**Figure 5:**
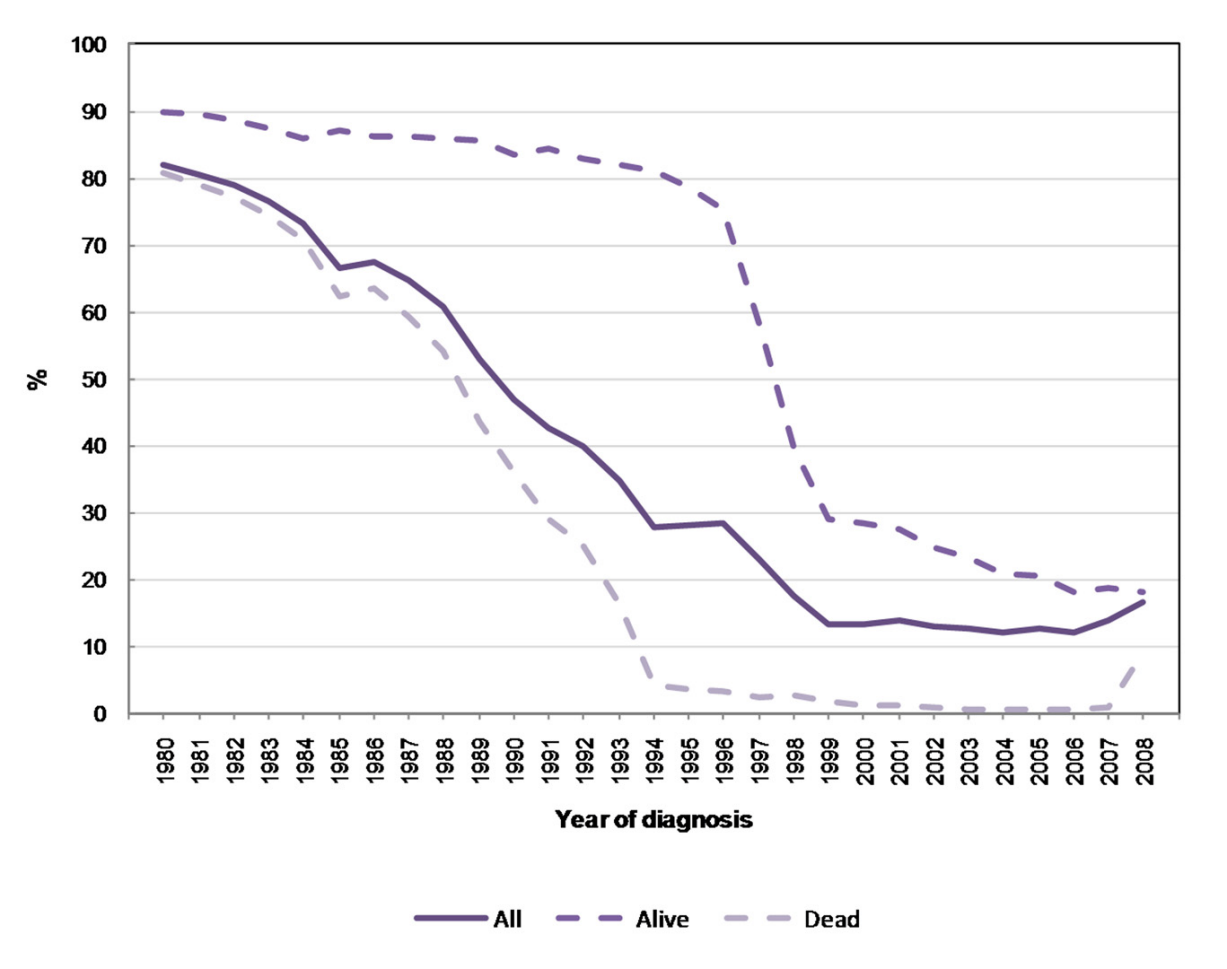
Survival from cancer in Aboriginals and non-Aboriginals, persons, males and females, 1999-2007.

Survival from cancer in Aboriginal people is worse: at 5 years, 35% of non-Aboriginal people in NSW have died from their cancer, compared to 47% of Aboriginal people (Figure 5, Table 6). When viewed as hazard ratios, adjusted for age and year of diagnosis and cancer degree of spread, Aboriginal people had an estimated hazard ratio of dying from cancer of 1.62 (p<.0001). In males, 51% of Aboriginal cancer cases have died of the cancer at 5 years compared to 36% of non-Aboriginal cancer cases. The corresponding male hazard ratio was 1.65 (p<.0001). For females the proportions surviving 5 years were 43% versus 33%, and the corresponding female hazard ratio was 1.59 (p<.0001). Cancers showing significantly lower survival in Aboriginal people include head and neck (males), stomach (females), large bowel and lung (males and females), breast and cervix. Of note, the largest Aboriginal/non-Aboriginal difference in 5-year cancer survival is in head and neck cancer among males (41% versus 67%), but in females this is identical (68%). Notable also are differences in survival from lung cancer: a similar proportion (91%) of Aboriginal males and females with lung cancer had died of it after 5 years, compared to 85% of non-Aboriginal male lung cancer cases and 81% of non-Aboriginal female lung cancers.

**Table 6:** Five-year cancer-specific survival (%) and adjusted^‡^ hazard ratios in Aboriginal versus non-Aboriginal people, males and females, NSW, 1999-2007, with 95% confidence intervals

Cancer site	Aboriginal		Non-Aboriginal	Hazard Ratio^‡^
Persons				
Head and neck	48.7 (38.4-58.9)^***^		65.6 (64.4-66.8)	1.90 (1.46-2.48)^***^
Stomach	17.1 (5.6-28.6)^*^		29.6 (28.3-30.9)	1.36 (1.01-1.82)^*^
Large bowel	50.7 (43.5-57.9)^***^		64.9 (64.4-65.4)	1.63 (1.35-1.97)^***^
Pancreas	4.4 (0.0-10.3)		6.7 (6.0-7.5)	1.32 (1.01-1.73)^*^
Lung	9.2 (5.4-13.1)^**^		16.3 (15.8-16.9)	1.47 (1.31-1.66)^***^
Melanoma of skin	90.6 (84.7-96.5)		90.4 (90.0-90.8)	0.98 (0.52-1.84)
All cancers	52.6 (50.3-55.0)^***^		65.4 (65.2-65.5)	1.62 (1.52-1.73)^***^
Males				
Head and neck	40.7 (28.2-53.2)^***^		64.7 (63.3-66.2)	2.17 (1.61-2.93)^***^
Stomach	19.9 (5.7-34.1)		29.6 (28.0-31.3)	1.18 (0.83-1.68)
Large bowel	47.9 (38.1-57.7)^*^		64.6 (63.8-65.3)	1.74 (1.36-2.22)^***^
Pancreas	8.0 (0.0-18.4)		6.2 (5.2-7.2)	1.19 (0.81-1.75)
Lung	9.3 (4.3-14.3)^*^		15.0 (14.4-15.7)	1.43 (1.22-1.67)^***^
Melanoma of skin	89.0 (80.1-97.9)		88.3 (87.7-88.8)	0.84 (0.37-1.90)
Prostate	77.6 (70.9-84.3)^***^		87.7 (87.3-88.1)	1.87 (1.37-2.57)^***^
All cancers	48.8 (45.5-52.1)^***^		64.1 (63.8-64.4)	1.65 (1.51-1.80)^***^
Females				
Head and neck	67.7 (52.4-83.0)		67.7 (65.5-69.9)	1.28 (0.71-2.30)
Stomach	11.4 (0.0-28.9)^*^		29.5 (27.3-31.8)	1.98 (1.15-3.40)^*^
Large bowel	54.6 (43.8-65.3)^*^		65.3 (64.5-66.1)	1.49 (1.11-2.00)^*^
Pancreas	8.5 (0.0-19.5)		7.2 (6.1-8.3)	1.46 (0.99-2.15)
Lung	8.9 (2.8-15.1)^*^		18.7 (17.7-19.6)	1.54 (1.30-1.84)^***^
Melanoma of skin	92.1 (84.3-99.9)		93.3 (92.8-93.8)	1.35 (0.50-3.64)
Breast	79.1 (74.0-84.2)^***^		87.8 (87.4-88.2)	1.83 (1.40-2.40)^***^
Cervix	59.0 (46.0-72.0)^*^		73.6 (71.5-75.6)	1.96 (1.30-2.96)^***^
All cancers	56.5 (53.4-59.7)^***^		66.9 (66.6-67.2)	1.59 (1.45-1.75)^***^

^‡^Adjusted for age and year of diagnosis, and cancer degree of spread.

^*^p < 0.05.

^**^p < 0.001.

^***^p < .0001.

### Sensitivity Analysis

If Aboriginals comprised 1% of cancer records with missing Aboriginal status missing, then the estimate of cancer incidence is 735 per 100,000 males and 481 per 100,000 females, with corresponding male and female SIRs of 1.16 and 1.07 and cumulated risks to 75 years of 1 in 2.5 and 1 in 3.4 respectively (Table 7). These estimates exceed those imputed here and of course increase with higher proportions of cancers with missing Aboriginality assigned as Aboriginal (cf. Tables 2 and 3).

**Table 7:** Sensitivity analysis: estimates of Aboriginal cancer incidence, NSW 1999-2007, assuming 1-5% of all cancers with missing Aboriginal status are Aboriginal

Proportion (%) missing assumed to be Aboriginal	Cases	Incidence^1^ per 100,000 (95%CI)	SIR (95% CI)	Risk to age 75, 1 in:
Persons				
0	2,318	493.4 (469.8-517.6)	0.95 (0.92-0.99)	3.3
1	2,700	587.7 (561.7-614.4)	1.11 (1.07-1.15)	2.9
2	3,083	678.6 (650.5-707.5)	1.27 (1.22-1.31)	2.6
3	3,466	771.6 (741.6-802.5)	1.43 (1.38-1.47)	2.4
4	3,849	864.5 (832.6-897.3)	1.58 (1.53-1.63)	2.2
5	4,232	955.3 (921.6-989.8)	1.74 (1.69-1.79)	2.1
Males				
0	1,201	595.2 (554.1-638.1)	0.97 (0.91-1.02)	2.9
1	1,437	734.7 (688.4-782.8)	1.16 (1.10-1.22)	2.5
2	1,649	861.6 (811.0-914.1)	1.33 (1.27-1.39)	2.2
3	1,888	1,000 (945.7-1,057)	1.52 (1.45-1.59)	2.0
4	2,106	1,130 (1,072-1,191)	1.70 (1.63-1.77)	1.9
5	2,335	1,261 (1,199-1,325)	1.88 (1.81-1.96)	1.7
Females				
0	1,117	419.7 (391.8-448.8)	0.95 (0.89-1.00)	3.8
1	1,263	481.3 (451.3-512.7)	1.07 (1.01-1.13)	3.4
2	1,434	546.9 (514.8-580.4)	1.21 (1.15-1.28)	3.1
3	1,578	606.0 (572.1-641.3)	1.34 (1.27-1.40)	2.9
4	1,743	672.8 (636.9-710.1)	1.48 (1.41-1.55)	2.7
5	1,897	735.0 (697.4-774.0)	1.61 (1.53-1.68)	2.5

^1^Directly age-standardised to 2001 Australian census population.

## Discussion

Based on an extensive assessment of NSW CCR records, Aboriginality recording rates were found to exceed 98% for NSW cancer mortality data from 1999 onwards, meaning that NSW Aboriginal cancer mortality occurring from 1999 can be reliably estimated without imputation. Aboriginal status recording in non-decedent cancer incidence data has improved markedly over 1996-99. Despite the improvement, imputation remains necessary for estimating cancer incidence and cancer survival in NSW Aboriginal people. For cancers overall, including decedent cases, unknown Aboriginal status affected 12-14% of cancer records over 1999-2007, but for cancers with good survival and/or little or no contact with the hospital system, missing Aboriginal status remains high (for example, ≈ 44% of all melanomas and 20% of all prostate cancers). With imputation, the estimated number of cancers in NSW Aboriginal people increased by 12-13% over that officially recorded. For melanoma and prostate cancer, imputation increased numbers of Aboriginal cases by 92% and 31% respectively.

Cancer incidence is 9% and 6% higher in NSW Aboriginal males and females than in all NSW males and females respectively, and corresponding cancer mortality is 68% and 73% higher. However, not all the Aboriginal cancer mortality excess is attributable to more advanced cancer at diagnosis: despite slightly lower incidence of localised cancer in Aboriginal people compared to non-Aboriginal people, mortality from localised cancer is significantly higher, and excess Aboriginal mortality from regional and distant cancer exceeds that expected from the underlying excess of incidence in these cancers. At 5 years following a cancer diagnosis, Aboriginal people are 34% more likely to have died from the cancer than non-Aboriginal people.

Notably, most of the excess of Aboriginal cancer incidence and mortality occurs in the oldest age groups and illustrates why different measures of incidence and mortality can be somewhat misleading. For instance, when directly age-standardised incidence and mortality rates are compared, as in a ratio of directly standardised rates in Aboriginal people to the total population (data for the latter not shown), then the excess incidence is 16-17%, substantially higher than the SIR-based estimates of 6-9%. However, excess mortality when estimated this way is 66-69%, the latter being closer to the SMRs reported here. Instead we have reported excesses in Aboriginal cancer incidence and mortality as indirectly standardised ratios and also as cumulated risk estimates which provide estimates less prone to artefacts of widely differing age distributions in the populations under comparison.

However, the age distribution of cancer incidence and mortality in Aboriginal people highlights a broader issue in Aboriginal health and mortality: the denominator populations of older Aboriginal age groups are small because they comprise those who have survived premature mortality from cardiovascular, endocrine, respiratory and other chronic diseases. Consequently, cancer has become a more prevalent cause of death in older Aboriginal age groups by default. Thus while the excess of morbidity and mortality rates from preventable cancers in Aboriginal people needs to be addressed, a substantial contributor to both incidence and mortality from cancer in Aboriginal people is excess mortality from non-cancer causes.

Possible explanations for the mortality excess over what may be expected from incidence or late stage at diagnosis include likely higher prevalence of co-morbid conditions in Aboriginal people which often precludes more effective cancer treatment, for example, organ resection. However, other factors likely also contribute to lower cancer survival in Aboriginal people. A study of lower survival from cervical cancer in New Zealand Maori women found that co-morbidity associated with cervical cancer explained only a moderate proportion of survival differences [8]. Access to effective treatment is an issue for many Aboriginal people where geographic remoteness, limited access to transport and accommodation are major issues [9,10], especially for treatment requiring extended periods of stay away from home and community, without support or facilities to cater for visits from large or extended families [11].

There is also evidence that many Aboriginal people are less likely to take up and adhere to some cancer therapies and treatments, often for cultural reasons, but also from negative experiences with the health system [12-14]. Importantly, a Western Australian study has found a deep sense of cancer fatalism to exist in many Aboriginal people, who perceive cancer as ‘a form of punishment resulting from some misdeed the person had done in the past’; as something that cannot be prevented; ‘equals death’; and is ‘god’s will’ [15]. Such cancer fatalism was found often to be accompanied by unrealistic expectations of treatment, and one consequence was that check ups following treatment are missed because the patient believes that they are cured [15]. It is thus probable that lower survival in Aboriginal people from cancers detected at the same stage and undergoing the same treatment as non-Aboriginal people may be due to lack of adequate follow-up and monitoring after treatment. Given that cancer treatment also often involves feelings of illness and unwellness, it is also probable that some Aboriginal people would perceive the treatment to be not working for them individually but doing harm. However, without definite knowledge of Aboriginal/non-Aboriginal co-morbidity and cancer treatment differences, including adherence, these explanations remain plausible at best.

However, an area in need of investigation is pharmacogenomic: to what extent can poorer cancer treatment outcomes in Aboriginal people be attributable to genetic factors that might influence response to anti-cancer drugs, including efficacy and toxicity. Ethnic differences in response to irinotecan (for treatment of a broad range of carcinomas including of the bowel and lung), gemcitabine (for treatment of solid tumours) and tamoxifen (for breast cancer) have been observed between Caucasians, Africans and Asians, and specific genetic factors isolated [16]. The genetic factors uncovered thus far do not account for all the variation by these ethnic groups, but personalised irinotecan therapy has been implemented in the US, Japan and Singapore [16]. Genetic studies of Aboriginal people in relation to common anti-cancer agents have yet to be conducted.

Our estimates of SIRs for Aboriginal people are higher than those recently estimated for Queensland [4], where male cancer incidence was estimated to be 28% lower than for all Queensland males (SIR = 0.72), and 15% lower for Aboriginal females (SIR = 0.85), compared to all-Queensland females. Most of the lower cancer incidence in Queensland Aboriginal people was attributed to lower rates of colorectal, prostate, breast and skin cancers than non-Aboriginal people.

In contrast, the present study found bowel cancer not to be significantly different in NSW Aboriginal compared to non-Aboriginal people. Melanoma SIRs for NSW Aboriginal males and females were estimated in the present study to 0.44 and 0.47, significantly higher than 0.06 and 0.07 for Queensland Aboriginal males and females respectively. While the Queensland study did not report absolute incidence rates for individual cancers, the counts of melanoma cases over a 10-year study period were about one third to half of the estimates in the present study for NSW covering a 9-year study period. The imputation process may have led to an over-estimate of of melanoma incidence in NSW Aboriginal people. However, the much lower melanoma SIR for Queensland would also partly be a consequence of higher melanoma incidence rates in the overall population of Queensland compared to NSW [17-19].

The present study found most cancers with excess incidence among Aboriginal people to be the same as those reported in the Queensland study. However, ovarian cancer incidence was found not to be significantly higher than in non-Aboriginal women, and head and neck cancers were over-represented in NSW Aboriginal people of both sexes, which in Queensland was limited to Aboriginal males [4].

Aboriginal people comprise 2.2% of the NSW population [1] and if a similar proportion were assumed to be representative of Aboriginal people in cancers with missing Aboriginal status, then from the sensitivity analysis (Table 7), Indigenous cancer incidence would be considerably higher than the estimates presented here. On this criterion, our estimates of Aboriginal cancer incidence here are somewhat conservative and suggest that Aboriginal people comprise <1% of cancers with missing Aboriginal status and consequently are more likely to have their Aboriginal status recorded in cancer registration than non-Aboriginal people.

Estimates of all-cancer mortality differentials in NSW Aboriginals were higher in the present study compared to the Queensland study. The latter estimated cancer mortality to be 28% higher in Aboriginal males than the all-Queensland male population (compared to 68% higher than NSW males in this study), and 47% higher in Aboriginal females than all-Queensland females (compared to 73% higher than NSW females in this study) [4]. Cancers contributing most to the Queensland excess were head and neck, lung, oesophageal, liver, and unspecified site (males and females), along with cervix and uterus in Aboriginal women. These cancers were also the main contributors to our estimates of excess cancer mortality in NSW Aboriginal people. While Aboriginal breast cancer mortality in the Queensland study was ≈ 25% higher than all Queensland women, in the present study it was estimated as 54% higher than all NSW women.

Our estimates of (relative) mortality from cancer in NSW Aboriginal people are similar to the 2006 NSW study by Supramaniam *et al.* for males, where Aboriginal male cancer mortality (for 1994-2002) was estimated to be 72% higher than all NSW males, but lower than the 65% excess estimated for Aboriginal females [3]. In the latter study, the authors adjusted for missing Aboriginality in NSW cancer mortality data by inflating observed Aboriginal cancer deaths by a factor of $\frac{100}{100\;-\;\%\;\mathrm{Aboriginality}\;\mathrm{missing}}$ and calculated the SMR using the resulting inflated numbers. While this procedure can produce plausible estimates of Aboriginal cancer mortality, the resulting sum of Aboriginal and non-Aboriginal cancer deaths would exceed that recorded for the total population.

In summary, the present study is the first to provide estimates of cancer incidence rates in the Aboriginal population of NSW, and the first to estimate NSW Aboriginal cancer mortality without imputation or other adjustment, the latter based on high completion of recording of Aboriginal status in cancer mortality data. As this is a population-based study in a setting of mandatory reporting of all malignancies diagnosed in NSW residents, it is not prone to biases originating from non-representative samples or from differential recording of cancers. A potential weakness of this study has been the assumption that Aboriginal status is correctly recorded when it is non-missing. Despite efforts to pro-actively record Aboriginal status in vital registration and in the NSW health system, this possibility underscores a need for some validation studies of Aboriginal status recording across the health and vital registration settings. More broadly, low rates of recording of Aboriginal status in cancers not commonly admitted to hospital (eg, melanoma) highlight important recording gaps in the health system that stem mainly from medical practitioners and pathology laboratories which do not record Aboriginal status.

## Conclusion

Despite some uncertainties around the extent of cancer incidence in NSW Aboriginal people, it is certain that NSW Aboriginal people suffer an unacceptable excess of preventable cancers, particularly of the lung, digestive tract and female genital tract. However, a large component of the excess of cancer incidence and mortality in Aboriginal people is occurring in older age groups of survivors, already depleted by mortality from non-cancer causes of death. Nonetheless, campaigns and initiatives directed at lowering risk factors for cancer in Aboriginal people clearly need to be more effective in addressing the significant social, economic, geographical and cultural barriers that prevent Aboriginal people from lowering their risk of acquiring preventable cancers. Maximising survival once cancer is diagnosed and treated in Aboriginal people may also need targeted therapies based on pharmacogenomic studies.

## Competing Interests

The authors declare that they have no competing interests.

## Authors’ contributions

SM drafted the manuscript, helped to conceive the study, participated in its design, and directed the statistical analyses. HY performed the statistical analyses. DB coordinated the study, participated in its design, and helped to draft the manuscript. All authors critically reviewed and approved the final manuscript.

## Pre-publication history

The pre-publication history for this paper can be accessed here:

http://www.biomedcentral.com/1471-2407/12/168/prepub
